# Everyday Life Theories of Emotions in Conflicts From Bali, the Spanish Basque Country, and the German Ruhr Area

**DOI:** 10.3389/fpsyg.2020.01339

**Published:** 2020-06-30

**Authors:** Robin Kurilla

**Affiliations:** Institute of Communication Studies, Faculty of Humanities, University of Duisburg-Essen, Essen, Germany

**Keywords:** emotion, culture, everyday life theory, conflict, emotion regulation, Bali, Germany, Basque Country

## Abstract

This article presents findings from a cross-cultural study on emotions in conflicts in Bali, the Spanish Basque Country, and the German Ruhr Area. The study had two aims: (1) to investigate the ways in which individuals make sense of how emotions, their expressions, and interaction conflicts are interrelated and (2) to compare the findings from the three regions. A particular interest was to explore and compare everyday life emotion theories. Ten semistructured interviews were conducted in each region. This method was triangulated with cross-cultural narrative interpretations and the task of relating emotion words to conflicts. The data were subjected to qualitative content analysis. The results show that partly different emotions were related to conflicts in the three datasets and that similar emotions may differ in antecedents, conceptual foundation, and behavioral consequences. Emotions similar to anger were commonly related to conflicts. In Bali, this emotion was mainly expressed through silence and rather hypocognated. The respective emotions received a deeper conceptual analysis and also served as conflict models in the other regions. Emotions similar to pride were related to the prolongation of conflicts in the Basque Country, considered causes of conflicts in the Ruhr Area, but were not related to conflicts in Bali. All three datasets show that the main indicators used to ascribe emotions to others are not facial expressions but subtle nuances and omissions of typical behavior and conventionalized signs. In the Basque Country, the emotions *respeto* and *confianza* form a continuum for the codification of interpersonal distance that produces different levels of expressiveness. These emotions act as a culture-specific socioemotional means of emotion regulation. The Balinese emotion *lek* has a similar function, as it neutralizes emotions similar to anger at their onset, acting as a substitute for deliberate forms of emotion regulation. All three datasets indicate that a hydraulic model is employed to conceptualize emotions, although the suppression of expressions is not pathologized in Bali but considered rather difficult to achieve. The communicative imaginaries of how emotions are experienced were surprisingly similar, with the exception that in Bali emotions are situated in the liver and described with a gustatory nomenclature.

## Introduction

Emotion and conflict appear together on the very first page of European history (Homer, 1928) and have been its loyal companions ever since. In fiction, arts, sciences, and humanities, the nexus between emotions and conflicts is a reoccurring motif. There is, however, a considerable amount of cultural differences regarding both emotions and conflicts.

This article aims at tracing the cultural differences of how people from the Spanish Basque Country, the German Ruhr Area, and Bali make sense of the relations among emotions, expressions, and interaction conflicts, focusing on everyday life theories of emotion. Conflicts have been chosen as a point of reference because they are part of the *conditio humana* and as such provide a common context of action with similar parameters in different cultural conditions with reference to which the performances of emotions become comparable. To further reduce the possibility of an ethnocentric bias, not two but three cultural contexts have been chosen as the base for comparisons.

From the range of the author’s knowledge of and access to different cultural contexts, two of the three regions (Bali and the Basque Country) were chosen according to the principle of maximum difference to account for high levels of cultural variation. The secondary, pragmatic reasons for the choice of the three regions lie in my institutional affiliation in Germany as well as my knowledge of languages, customs, and cultures. The study’s preconceptions were derived from ethnographic observation, participation in everyday life of each field for a period of at least 1 year, and ethnographic literature. It was assumed, among other things, that the cultural contexts comprise sufficient variation for a cross-cultural study of emotions, emotionally guided models of conflict, conflict-related action strategies, and ways of regulating emotions and conflict.

The classical ethnographic literature on Bali depicts life on the island as an absolute harmony between individuals and society. Bateson (1999) did not encounter processes of symmetric schismogenesis in Bali and describes the society as system in a steady state. According to Geertz (1987a), Balinese people are masters of disguise and ambiguity who almost compulsively avoid open conflicts. Considering their lack of expressiveness, Mead (1942) ascribes a “schizoid” character to the Balinese. To Geertz (1987b), Balinese people owe their compliance with a strict etiquette of expressiveness to their fear of demons and to the emotion *lek* that Geertz (ibid.) translates as stage fright. This translation implies that the Balinese are constantly acting as on a theatrical stage to control expressions of emotions. The description of the Balinese strict etiquette of avoiding the expression of so-called negative emotions has not been challenged until today (Beatty, 2005, 2014; Howe, 2005; Haley and Richeport-Haley, 2015). Through her studies of a predominantly Muslim community in Northern Bali, however, Wikan (1987, 1989, 1990) came to the conclusion that Balinese people do not behave like actors in their own understanding. According to Wikan, the expression etiquette is rather maintained through fear of others and of sorcery. For fear of negative consequences of their own expressions, Wikan assumes, Balinese people engage in a constant procedure of “managing the heart,” that is, in emotion regulation to keep their expressions within an accepted range.

In contrast to Bali, interactions in the Spanish Basque Country can be prone to emotional outbursts. Loud conflicts, however, tend to disappear rather quickly without permanently damaging interpersonal relations. In the German Ruhr Area, in turn, open conflicts are not uncommon but mostly not as expressive as in the Basque Country and tend to last longer. A common reason for conflicts in public is the transgression of norms. If anyone ignores a red traffic light and just walks over the street, he/she is likely to be criticized by bystanders, which is very unlikely to happen in the Basque Country. This sketch of differences between the three cultural contexts is sustained by participant observation and provided the author with sufficient confidence that a comparison would be fruitful.

The study is based on the assumption that emotions are social phenomena in two respects: (1) they serve social functions, particularly on the interaction level; (2) they have a social origin. The first assumption is based on Bühler’s (1978) pragmatist account according to which emotions as well as their expressions facilitate the coordination of social action. From this viewpoint, the expression of anger in conflicts, for example, appears as an instrument or means of conflict, aimed at guiding the behavior of others. The assumption of emotions being social fabrications builds on the epistemological implications of social constructivism and not so much on the regulation of emotions through “feeling rules” in the sense of Hochschild (1979). It is simply assumed that “emotions can only be fully understood as part of the culture as a whole” (Averill, 1980) and are thus culturally and historically variable. Evidence for the social nature of emotions is provided by, for example, Levy (1973), Luhmann (1983), Lynch (1990), Suryani and Jensen (1993), Stimilli (1996), Mees and Rohde-Höft (2000), Schröder (2004), Rousseau (2009), Harbsmeier and Möckel (2009), Sundararajan (2015), and Crivelli et al. (2017).

The design of the study was developed to suit three requirements: it should (a) pay attention to actual social practices, not only linguistic models; (b) be faithful to the inside views of individuals and collectives; and (c) provide a basis for the comparison of different cultural contexts. In the following three paragraphs, I will briefly elaborate on these points, indicating some of the research this study critically builds upon.

Studies on linguistic localizations of emotions in different parts of the body are of interest for the study as long as they do not ignore social practices that differ from linguistic descriptions. Gerber (1985), Heelas (1986), Harré and Finlay-Jones (1986), Wierzbicka (1995), and Omondi (1997) trace how emotions are localized in different parts of the body through language. Lakoff (1987) and Kövecses (2000) pay special attention to the use of metaphors. These studies, however, are prone to confuse two logical levels or to “confound the map with the territory.” It is rather unlikely that social practice can be described or even planned and exercised with the help of propositions, reflective discourse, and linguistic models alone. As a consequence, research has to pay attention to emergent social processes, as they might take other paths than their linguistic depictions would suggest.

Ethnographic studies provide detailed descriptions of the role of emotions in conflicts in different cultural contexts that go beyond linguistic models of social practices and highlight cultural features. Rosaldo (1980, 1997), Lutz (1998), Röttger-Rössler (2008), and Busher et al. (2018) surely give faithful accounts of emotions and their relations to conflicts in the fields they studied. Their work does, however, raise questions concerning the comparability with other fields.

Nonethnographic studies of the emotion dynamics of conflicts often disregard the inside views of individuals and collectives but bear an advantage in view of comparability. To Retzinger (1991) and Scheff (1997), unacknowledged or bypassed shame turns into anger and fuels conflicts. This model renders the socioemotional dynamics of certain conflicts comparable, but it is immune to counterevidence stemming from the inside views of individuals. There are a number of studies on intercultural communication, negotiation, and conflict resolution that suffer from a similar problem. Allred et al. (1997), Rahim et al. (2002), Bell and Song (2005), Maoz and McCauley (2005), Kopelman and Rosette (2008), Zhang et al. (2014), and Luomala et al. (2015), among others, use a quantitative and/or experimental approach to investigate cultural differences in the relations among emotions and conflicts. Categories are not derived from the data but determined *ex ante*. This results in an increased degree of comparability but, at the same time, also in a lack of faithfulness regarding the inside views of collectives and individuals. If the researchers do not know the relevant categories, the results are necessarily biased, maybe ethnocentrically. Researchers following this approach can only reap what they sow. Moreover, the categories that quantitative cross-cultural research on emotions in conflicts commonly projects onto its subjects entail objectivistic notions of culture. It is not necessarily clear that the differentiation of collectivistic and individualistic cultures or independent and interdependent selves makes a difference in social practice. Similarly, it remains doubtful that cultures can be identified with national or continental borders and that the talk of “Western” and “non-Western” cultures has any analytic value considering nowadays’ global social and cultural interconnectivity and hybridity.

To reach its goals, the present study faced the task of balancing the antagonism between faithfulness and comparability. To tackle this methodological challenge, a qualitative approach has been chosen that generates data from three different cultural contexts. In addition, local narratives on emotions and conflicts were interpreted from participants of other cultural contexts to highlight differences in sense-making in a cross-cultural way. Like Frevert (1991) in her study of the historical development of duels in European history and Röttger-Rössler (2008) in her ethnographic research, I pay special attention to cultural interaction models and their influence on social practice and emotions without disregarding that they are placed on two different levels of analysis. Following Simmel (1908) in view of jealousy and Luhmann (1983) regarding love, however, some emotion terms receive a double consideration—as referring to emotions and to models for social action that are employed to structure social intercourse and fabricate emotions.

## Materials and Methods

The study has a two-fold general aim: (1) to investigate the ways in which individuals make sense of how emotions, their expressions, and interaction conflicts are interrelated; (2) to compare the findings from the Basque Country, the Ruhr Area, and Bali. A particular interest was to explore and compare everyday life theories of emotion.

Hypotheses were developed during research in orientation on an abstract theoretical frame that allows for specifications in a variety of empirical fields. In the process of hypotheses generation, the theoretical frame was concretized and tested with empirical data. This qualitative methodology is best understood as “empirisch begründete Theoriebildung” in the sense of Kelle (1997), who critically builds upon not only Glaser and Strauss (2009) and Strauss and Corbin (1998), but also Popper’s (2002) conception of a “quasi-inductive evolution of science.” From the viewpoint of quantitative methodology, approaching the fields in this manner can serve exploratory purposes.

### Methods

In order to account for cultural differences but simultaneously provide for comparability, I conducted semistructured qualitative interviews with a special focus on personal narratives on emotions in conflicts. The interviews were triangulated with two other methods. Participants also related emotion words to conflicts and were asked to interpret the narratives from the other two cultural contexts and, except for Bali, their own.

The main function of the interviews was to stimulate seminatural talk on emotions in conflicts in view of a variety of topics detailed in the interview guide (Table 1). The interview guide was modified and elaborated during research to include topics that were not expected prior to the study and thus enhance theoretical saturation in the sense of Saunders et al. (2018). A special section for Balinese people was also included because of preconceptions derived from the literature and further elaborated in view of unexpected findings from the interviews. The order of questions was subject to interaction dynamics and did not necessarily follow the order envisioned in the guide. At the beginning of most interviews, however, participants were asked to tell stories from their daily lives in which emotions and conflicts played major roles. Interviewees detailed the roles emotions play in workplace conflicts, marital quarrels, conflicts among friends and family, politically driven conflicts, public conflicts in a village, quarrels during gambling, conflicts of opposing fractions of a youth organization, and so on.

**TABLE 1:** Interview guide.

**Introduction**
Objective of the interview: Research on emotions in conflicts from the viewpoint of communication studies
Timeframe: approximately but not reduced to 45 to 90 min (depending on the willingness of the participants and the productivity of the conversation)
Details: The focus lies on interaction conflicts with two or more participants – face to face, on the phone, at home, at work etc. Political and group conflicts have a secondary status. The interviewees’ personal experience is at the center of attention.
Explanation of the procedure, the recording devices, and the confidential handling and storage of personal data
Informed consent

**Phase I: Conflict stories**
Conflicts are a delicate subject that can be very disruptive but, at the same time, is also very common. What conflicts do you know from your own experience? It does not make a difference whether you were involved in those conflicts yourself or one of your acquaintances. What happened? What was the trigger? How did participants treat each other? When was the conflict over? Was there a winner and a loser?
Examples to stimulate the conversation: conflicts among couples, at the workplace, within the family, in literature, film, and television.
Do you know more of these stories that are either very similar to or very different from this one?

**Phase II: Conflict types**
You have told me about (number) conflicts. Are there more typical conflicts? We had the couple conflict, the conflict among friends in front of a group, the conflict with your daughter…
In what way do these conflicts differ from each other?
How does the behavior of individual participants differ regarding situations and roles?
What has to be avoided in conflicts? Who has the right to do what?
You surely know typical patterns of conflict: A father prohibits his daughter to do something, and she runs into her room and slams the door. The father’s wounded pride leads him ground his daughter. The daughter starts to weep, which might lead the father to have a bad conscience… Please describe such a pattern.
How would you describe the participants of different kinds of conflict? What are their characteristics?

**Phase III: Conflict strategies and emotions**
When there were emotions involved in your conflicts, which emotions were these?
How did the emotions make themselves noticeable? Did you have difficulties in communicating them? Did you even want that?
How do you quarrel? How does one avoid quarrels? Does it make sense to avoid quarrels?
Do you and others tell each other what you think and feel during a quarrel?
When does it make sense to communicate, to hide, to camouflage etc. emotions in conflicts? Are there any, home recipes’ for different situations?
How does the role of emotions change in the different conflicts we have been talking about? If you were really upset about something, you wouldn’t let yourself go at work in the same way you would among friends, would you?
What types of people who behave always similarly in conflicts do you know? The fear biter, the master of avoidance…
What types of emotional conflict strategies in which emotions are openly communicated do you know?
What persons show their emotions in what manner? How do people react to that?
How does one handle emotions in conflicts if these are not supposed to surface? How does one recognize if someone suppresses his or her emotions?
How does one react in what situation in what conflict in what role to a certain communicated emotion?
What do you do if you find that an emotion is not justified?

**Phase IV: Forms and vocabulary**
Different words refer to interaction conflicts and may also designate different kinds of conflict. Take, for instance, “quarrel,” “disagreement,”“marital row”, etc. Which ones do you know?
In which situations do these forms occur? A marital row in a three star restaurant, at home, or at work – does that make a difference?
There are also typical participants in those conflicts. It is rather unlikely, although not impossible that a quarrel at the workplace takes place between father and son, which would rather be typical for a family quarrel. Who are the participants in the case of conflicts of type x, who are the participants of a conflict of type y? What strategies do participants apply? How do they communicate?
Please attribute emotions to individual participants and justify your decision.
There are surely more signifiers of emotions that are related to conflicts. Which ones are there?
How are these individual emotions related to conflict strategies? To make this simpler, let’s start with the types of conflict in specific situations with different participants that we just talked about. What emotions would you attribute to person *x* in conflict y and situation *z*? How do participants communicate or suppress their emotions?
Why? What for?
We have now a list of emotion words, conflict forms, actors, and communication strategies. Please tell me how this all fits together. In a given conflict, which emotion triggers what behavior in which actor, or which behavior triggers which emotions?

**Phase V: Emotion dictionaries**
Lists of emotion terms derived from three emotion dictionaries were presented to the participants. The task was to specify if the individual emotions on the lists was a cause, an instrument, or an outcome of a conflict.

**Special questions for Balinese people**
How can one explain a behavior that is not guided by intentions?
What role does nonverbal action play in conflicts – is it more an expression or an instrument of interpersonal guidance?
Who is in control of the body?
Do people rather express their emotions in quarrels or do they rather complain about the other?
What is the authentic? A seemingly fake smile or the emotion that might be hidden by the smile?
Why do Balinese people smile so often and hardly show aggressive behavior?

Members of each field interpreted the stories from the other fields and sometimes their own cultural context in order to highlight cultural differences in interpretation. This method required an iterative procedure. The first interviews were recorded in the Basque Country when no data were available from the other two regions, so that no interpretations of narratives could be included at that time. Before the interviews were recorded in Bali, two Basque stories about envy and jealousy had been obtained from an informant and discussed with another person in order to test if the interpretation made sense for others. As there were still no data from Germany, one story was obtained from German news media and another one from a personal account of a German acquaintance. Balinese participants were asked to interpret these four narratives from their own points of view. Three additional stories were extracted from the Balinese data before the interviews took place in the Ruhr Area. German participants interpreted the two Basque, two German, and three Balinese stories (Table 2). As the original participants from the Basque Country had not interpreted any stories, additional interviewing took place in the Basque Country with seven new and one of the original participants. These interviews focused exclusively on the interpretation of the seven narratives.

**TABLE 2:** Stories on emotions in conflicts.

**Basque story 1**
A couple goes shopping. Suddenly, the woman starts to shout: “Why the heck do you have to look at other women all the time? You stare at them and even smile once you notice that they like being looked at by you!” The man tries to justify himself by stating that he only wanted to be nice when he realized the woman had smiled at him. But it seems to be too late for an excuse: “You nasty piece! How could you have noticed that she was smiling, had you not looked at her first?”
What happened to the woman?
What did she feel?
Why can’t the man look at other women?
How should he react now?
Was the woman’s final utterance appropriate?
What type of utterance was it, and to what consequences do such utterances lead usually?
What will happen next in view of the utterance?
How should the man be feeling?
Are such situations common here?

**Basque story 2**
Two girls lie on the beach. A young man comes by, kisses both girls on the cheeks and then almost exclusively talks to one of the girls. The other girl remains silent during the conversation. When the man departs, his interlocutor seems rather pleased. She enthuses about him, saying he was so nice and good-looking. The other girl, however, does not to share the same opinion: “I have met this guy before, and he did not make a good impression on me from the start. He does not look like somebody you can trust.” But this does not bring the other girl’s enthusiasm to an end: “Aha. Anyway, I guess I will find out myself when I meet him this evening.” Thereafter the other just remarks that she only wanted to warn her friend and changes the topic abruptly.
What happened to the girl that kept silent while her friend was conversing with the young man?
Why didn’t she say anything?
What did she feel?
Why didn’t she share her friend’s enthusiasm?
What could be her hidden intention?
Can you identify a hidden conflict and the corresponding behavior in the story?
What did the girl feel when she told her friend that the man could not be trusted?
What did the silent girl feel when her friend remarked she was going to meet the man in the evening?
Are such situations common here?

**German story 1**
Two young men with a migration background travel by metro. An old man tells them that they are not allowed to smoke in the metro. Unimpressed by this, the men insult the old men who stands up to take another seat. When the old man leaves the train he provokes the two implicating their descent: “You are the people (Volk) that causes us problems here. Subsequently, the two men pursue the old man and almost beat him to death.”
What die the old man feel when commenting on the ethnic origin of the two?
Why did he talk to them like that?
What does it mean if someone talks to others like that and to what consequences does it lead?
How did the two men feel when they were addressed like this by the old man?
Why did they almost kill the old man?
How would one react normally in such a situation?
Are such situations common here?

**German story 2**
A 12-year-old boy burns a sticker that hangs at the glass walls of a bus stop with a lighter. A middle-aged man passes by and shouts at the boy. The boy says: “I have not placed the sticker there, but I am cleaning the wall with my lighter.” The man hits the boy into his face. Coincidently, a slightly older friend of the boy comes by and asks what is going on. The man just keeps screaming/shouting. The boy’s friend tells the man that he should treat him more respectfully (addressing him as “Sie” instead of “Du”). The man hauls off to hit the boy’s friend. But the kid is faster, getting out a can of tear gas of his pocket to threaten the man. Seemingly surprised, the man turns around and walks away, still shouting and complaining about nowadays’ youth.
What does the man feel when he sees the boy burning the sticker?
Why does he hit the boy?
Would one normally react like that?
What does the man feel after the boy’s excuse?
Why became the friend of the boy interested in the incident?
What did the friend feel and why?

**German story 2**
Why didn’t the man also hit the friend of the boy?
What did the man feel when he refrained from hitting the boy?
Why was the man still shouting when he left the scene?
Are such situations common here?

**Balinese story 1**
Two men meet at a cockfight. One of them has just lost a bet. The other one asks him what he was doing at the cockfight. He had thought he was long dead because he had not seen him in a long time. Subsequently, the other man starts a discussion/turns around and walks away.
What does the man feel who has just lost a bet?
What does he feel when he hears that the other man thought he was dead?
Was the utterance about his death appropriate?
Is it normal here that people ask whether one was not dead already?
How could the story continue?
Are such situations common here?

**Balinese story 2**
A father loses his money gambling. When he comes home, he reprimands his wife who cannot comprehend how her husband could gamble with let alone lose the tuition fee of his children – without consulting her fist. Despite his rough behavior, she feels for her husband and tries to understand him. Nevertheless, the couple separates during the ensuing quarrel. After a while the wife returns to the shared household because her husband promised her that he will gamble less, find a job, stop disregarding his children, and stop to harm them through his behavior.
What does the father feel when he loses the money?
Why does he reprimand his wife?
How does his wife feel when he reprimands her?
What does the man feel when he reprimands his wife?
What does the woman feel when she finds out that her husband lost the tuition fee of their children through gambling?
Why does the couple separate temporarily?
What does the woman feel when she goes back to her husband?
What does the man feel when he promises to become a better person?
How does the story continue?
Are such situations normal here?

**Balinese story 3**
One of the two youth organizations of a village caricatures a politician by building a statue of him from papier-mâché that depicts him as a monster. When the politician finds out about the work on the statue, he complains about it and tells the members of the organization that they cannot continue their work and have to find another subject. As the politician also holds the dignified status of a priest, some members of the organization agree with his demands. In the end, the incident appears to escalate. People threaten with physical violence and almost recur to it when suddenly the police enter the scene, probably notified by neighbors sympathetic to the politician.
What does the politician feel when he finds out about the statue?
What accounts for the different reactions among members of the youth organization?
How do the different camps within the organization feel? Why?
What does the politician feel when he finds out that the majority of the organization is not on his side?
How does the politician feel when he sees himself confronted with physical violence?
What could have been his reaction to violence?
Why is violence used (as a threat)?
How did participants feel when the police arrived?
Are such stories common here?

The third method employed in the frame of the interviews consisted in conceptually relating emotion terms to conflicts. These terms were derived from Heider’s (2006) maps of emotion clusters for Indonesian, Marina and López (2007) “diccionario de los sentimientos” for Spanish, and the “Affektives Diktionär Ulm^1^” for German. When participants did not find any relations between emotions and conflicts from a reflective point of view, they were given the options of emotions being a cause, a result, or a means of conflict to stimulate the conversation. Especially the Indonesian and the German emotion words had to be reduced to a smaller number with the help of informants from the regions because they were originally too numerous to be handled in the interviews without overwhelming the interviewees.

### Participants

In each of the three fields, I conducted 10 interviews. One interview of each field could not be processed due to technical difficulties, so that only nine interviews were subjected to analysis [Ruhr Area: *n* = 9, 55.56% female, mean = 29.45 years, SD = 5.14 years, range = 23–39 years, higher education^2^ (HE) = 66.67%; Basque Country: *n* = 9, 55.56% female, mean = 28.55 years, SD = 5.85 years, range = 21–40 years, HE = 77.78%; Bali: *n* = 9, 11.11% female, mean = 34 years, SD = 14.16 years, range = 20–63 years, HE = 33.33%]. In the Basque Country, I conducted eight additional interviews that focused on the interpretation of narratives (*n* = 8, 37.5% female, mean = 29.38 years, SD = 4.74 years, range = 23–40 years, HE = 37.5%). Because of the study’s cross-cultural orientation, factors such as milieu were set aside regarding the selection of interviewees. A relative heterogeneity of sociodemographic criteria guided the search. In order to include an outsider’s perspective, one person with a migratory background was recruited in each field. Public postings, word of mouth, and spontaneous inquiries helped to facilitate the recruitment process. Local dispersion served as an additional recruitment criterion in Bali, as more traditional perspectives were expected in the rural areas than in the urban and touristic areas.

### Procedure

Participants received printed and verbal participant information, and their full and informed consent was obtained. Informal settings not uncommon to the participants’ life worlds were chosen for the interviews in all fields to facilitate seminatural communication. The interviews were conducted in Spanish, German, and Indonesian. Whereas in the Basque Country and the Ruhr Area all interviews took place between the interviewee and the interviewer as sole participants, the personal context sometimes interfered in the interviews in Bali. Instead of limiting or impeding the remarks of third parties, they have simply been accepted to avoid interference with local habits of communication. Small mp3 devices recorded the interviews in an unobtrusive manner. The interview guide was handled flexibly in the sense that its chronological order remained open to communication dynamics. Because of these dynamics and individual communication styles, the interview lengths varied between 30 and 120 min, differing from the initially estimated time frame of 45 to 90 min.

### Analysis

The recorded audio documents were transcribed with the help of the software EXMARaLDA. The author created a simplistic notation tool to highlight salient linguistic features such as silences of different lengths or peaks of voice.

The author read the transcripts multiple times while picturing the interview situation. This first step of analysis led to the formulation of content paraphrases for each transcript. In a second step, the paraphrases were again paraphrased and canonized. In order to ensure that the second paraphrasing did not estrange the results from the transcripts, the transcripts were used to validate the second paraphrases. Individual topics were condensed to broader themes. Actual theme clusters were constructed in the third step of analysis. The nature of the data made it necessary to allow for individual themes to appear in various clusters.

After the clusters for each interview were completed, the clusters of all interviews from one field were interrelated. Special attention was paid to disconfirming cases to improve the validity of the findings. To validate the soundness of interpretation on this level of analysis, the original transcripts were consulted. The thereby emerging clusters provide the foundations of the results concerning each field as a whole. On the basis of these results and the paraphrases that helped generate them, the three fields were compared to each other. I am confident that the data have been sufficiently interpreted regarding the theoretical frame of the study. Saturation may, however, not have been reached regarding all possible topics that might still emerge from the data but do not concern the focus of research. I discussed the results with different individuals from each cultural context, which strengthened my confidence in the findings.

A communication-theoretical conceptual framework guided the analysis of empirical data. From the viewpoint of an orthodox qualitative approach, this might resemble an attempt to imitate the methodology of quantitative research. Lamnek (1995), for example, refers to this point in his critique of Mayring’s content analysis. The described way of analysis is indeed loosely based on Mayring’s (2010) content analytical procedures. The conceptual framework, however, does not consist of a fixed set of hypotheses that were developed prior to the empirical study. It is rather situated on an abstract level that leaves sufficient variability to be specified in view of cultural variation. Such an explication of theoretical assumptions prevents researchers from the epistemologically very questionable notion that knowledge emerges purely from data.

## Results

A thorough discussion of all details and implications of the results would exceed the scope of this article; see Kurilla (2013a, b) for a contextualization of the results within a historiographic and communication-theoretical frame. This section focuses on field-specific relations among emotions and conflicts, forms of emotion regulation, and everyday life theories of emotion from each field. Table 3 shows all theme clusters that emerged from the data. For each of the three cultural contexts, I start with individual emotions and their relation to conflicts and come then to broader themes such as emotion regulation and everyday life theories of emotions. Whenever direct speech is reported, it refers to what individual participants said during the interviews and, at the same time, is paradigmatic for a given cluster. It is, however, not representative in the sense of quantitative research but rather depicts what some individuals believe. The section concludes with a comparison of the findings.

**TABLE 3:** Theme clusters from each field (with approximate translations of the emotion words).

**The Basque Country**	**Bali**	**The Ruhr Area**
**Emotions in conflicts**		
angustia (anguish)	amarah (anger)	Angepisstsein (pissed off/to be pissed on)
ansiedad (anxiety)	benci (hatred/dislike)	Angst (fear/angst)
celos (jealousy)	emosi (emotion)	Ärger (anger)
confianza (trust/confidence)	hormat (respect)	Aufgebrachtsein (to be upset)
enfado (anger)	iri (envy)	Aufregen (to be upset)
envidia (envy)	jengkel (annoyance)	Ehre (feeling of honor)
ira (rage)	kasihan (pity)	Eifersucht (jealousy)
miedo (fear)	kecemburuan (jealousy)	Enttäuschung (disappointment)
Nerviosismo (nervousness/edginess)	kecewa (disappointment)	Frustration (frustration)
orgullo (pride)	kesal (grudge)	Hass (hatred)
rabia (rage)	malu (shame)	Mitleid (pity)
rancor (grudge)	takut (fear)	Neid (envy)
respeto (respect/regard)		Respekt (respect)
tristeza (sadness)		Sauersein (to be sour)
		Scham (shame)
		Sich-verarscht-Fühlen (to feel fooled)
		Stolz (pride)
		Stress (stress)
		Trauer/Traurigkeit (sadness)
		Vertrauen (trust/confidence)
		Wut (fury/rage)
**Other clusters**		
Gender stereotypes	Gender stereotypes	Gender stereotypes
Regulation of emotions and conflicts	Regulation of emotions and conflicts	Regulation of emotions and conflicts
Everyday life theories on emotion, conflict, and communication	Everyday life theories on emotion and conflict	Everyday life theories on emotion and conflict
Emotion and context	Emotion and context	Emotion and context
Tears and crying	Expression vs instrument	Expression vs instrument
	Process gestalts in conflicts	Process gestalts in conflicts
	Authenticity	Proximity-distance
	Change of emotion through aging	The others
	Mediated conflicts	Phenomenal descriptions
	karma	Emotion sequences
	ilmu hitam (black magic)	Public vs private
	Memedi (devil)	
	hati (liver)	

### Emotion Theories in the Spanish Basque Country

The 19 clusters that emerged from the Basque data comprised 14 different emotions, namely, *celos*, *confianza*, *enfado*, *envidia*, *ira*, *ansiedad*, *angustia*, *miedo*, *nerviosismo*, *orgullo*, *rabia*, *rencor*, *respeto*, and *tristeza*. The remaining clusters concern gender stereotypes, regulation of emotion and conflict, everyday life theories of emotion, and so on.

#### Confianza and Respeto

*Confianza* (confidence, trust, faith) and *respeto* (respect) are the emotional manifestations of the continuum of interpersonal proximity and distance. The more *confianza* people feel toward each other, the closer their relationship is. Some interviewees stated that true *confianza* exists primarily between friends rather than romantic partners. The duration of relationships projected into the future is an indicator of *confianza*. Consequently, the family and old friends are the main addressees for this emotion. *Confianza* enables individuals to communicate more openly with each other. From this angle, *confianza* is comparable to Scheff’s (1997) conception of pride as the emotional manifestation of a secure social bond. Unlike this concept of pride would suggest, however, the interviewees correlated high levels of *confianza* with a particularly harsh and loud conflict style. To put it another way, *confianza* enables people to express their emotions more freely. Accordingly, quarrels with family members, close friends, and romantic partners are considered more expressive. Screaming and shouting are more common in these relationships than in conflicts at the workplace. These findings correspond to Simmel’s (1908) depiction of close relationships as the base for more intense conflicts.

*Respeto* is the emotional opposite of *confianza* in the sense that it codifies interpersonal distance. It helps to regulate emotions in formal contexts. *Respeto* is particularly connected to interpersonal relations at the workplace and in the general public.

At the same time, it acts as the source of conflict in some cases. Various interviewees described how a lack of *respeto* (*faltar al respeto*) is held responsible for aggressive conflict styles where anger and rage are freely expressed.

“Entonces las broncas siempre eran […] porque una persona tiene que respetar a la otra, y en el punto en que esa persona deja de respetar, yo salto.” “Entonces a mi me pone muy nerviosa cuando, si alguien […] me conoce y sabe que yo tengo unas costumbres y que yo las respeto, esas costumbres de las otras personas […] Eso es, que me, que me fallen, entonces grito. Y yo tengo unas peleas de gritos, que me ataco, vamos. […] ‘pero que… que eso es mi intimidaaad,’ ‘!‘que respetes!’ Entonces yo de esas broncas he tenido muchas. Siì, no son conflictos de, no sé, no son conflictos graves pero me enfada mucho, cuando alguien no respeta cosas, que yo creo que son, pues, de la viida de cada uno, […] me pone muy nerviosa o discuto mucho […].”

These examples indicate that a lack of *respeto* is ascribed to people who transgress personal boundaries of intimacy or distance. Other symptoms of a lack of *respeto* are insults and violence. At the same time, *respeto* acts as a social regulation of human relations with emotional manifestations. *Respeto* as an emotion seems to assist in the personal downward regulation of violent emotions and recognition of interpersonal boundaries.

#### Orgullo

According to most interviewees, *orgullo* (pride) propels the escalation of conflicts and acts as an obstacle of reconciliation. On the one hand, *orgullo* is considered as the reason to include more and more issues into conflicts; on the other hand, *orgullo* stimulates the desire to win conflicts. When *orgullo* is ascribed to others, this may render the own willingness to initiate acts of reconciliation more likely because the others cannot be expected to do so. For this reason, *orgullo* can serve as a strategic façade that leads others to accept one’s own claims. From the inside view of a person who actually feels *orgullo*, however, this emotion cannot just be “swallowed”:

“[…] unos valores que te marcas tú..… ¿Sabes? que si yo creo que tengo razón en una cosa y la veo totalmente clara, y otra persona me me dice que no es así.… Tú puedes llegar a entender unas cuantas cosas, pero ese […] círculo que está ahí que tú consideras tu orgullo, tus valores, tu honor… es algo que es difícil de […] desmentir, decir que eso […] no es así. Algo como que lo que tú […] lo entiendes como tus pilares como persona[.]”

According to this statement, *orgullo* is related to personal values as the pillars of the individual. To injure one’s *orgullo* is thus an affront to the core of the self. Because of its relations to values, *orgullo* bears also positive connotations despite its capacity to impede reconciliation. People seem to appreciate others who are able to become *orgulloso* to a certain degree, as it shows that they actually stand for their personal values. These twin faces of orgullo were confirmed by a reviewer of the article who differentiates between “ser orgulloso” and “estar orgulloso,” the latter of which bearing more positive connotations. Coincidently, the other reviewer pointed at the double meaning that “pride” seems to have also in English. When it is related to personal accomplishments, it appears to be socially endorsed, whereas it seems to evoke negative connotations when it “gets in the way of interpersonal relationships.” Unlike Scheff’s (1997) concept of pride as a manifestation of secure social bonds that helps to act out conflicts openly in a rational and respectful manner without being corrupted by bypassed shame, at least the negatively evaluated face of *orgullo* is rather prone to stimulate and prolong emotionally charged conflicts.

#### Tristeza

Participants described *tristeza* (sadness) as a means to soothe conflicts. One’s own sadness may lead individuals to hold back strong opinions and violent emotions that are directed at others. Conversely, the *tristeza* of others may elicit the same effect in oneself. The latter makes it possible that the expression of *tristeza* is strategically employed in conflicts to escape one’s own social or communicative obligations at least temporarily.

“Es un mecanismo de defensa […] para evitar una discusión o para no asumir una culpa cuando veo que no tiene ninguna justificación y llora es como intentar sacar la pelota de su tejado. Yy entonces pues bajo mucho el tono para que para que no sea por la agresividad del lenguaje lo que está causando pero si tengo alguna reclamación o tengo ae un desacuerdo en otros términos más suaves o lo que sea, o cambio el tema o o cierro el tema y vuelve a él más suavemente cuando se pueda, pero no me no suele funcionar conmigo como para evitar el problema. Se puede aparcar pero no se evita.”

One interviewee compared the *tristeza* of others to a powder keg that has to be handled with care. Another interviewee stated that her *tristeza* might lead to conflicts when it is transformed into *rabia*.

#### Rabia

Besides slamming doors, shouting, and screaming, *rabia* (rage) is counterintuitively often expressed through tears, just like *tristeza*. From the perspective of one interviewee, *tristeza* and *rabia* are closely connected and not always separable in experience. Transformations from *tristeza* to *rabia* seem to be expectable in certain interaction dynamics according to two interviewees. Other interviewees related *rabia* to helplessness:

“[…] la rabia […] un sentimiento ya de desesperanza […], es algo desesperado. O sea la rabia […] está asociada a un sentimiento de impotencia, entonces, en el momento que estés, te sientas impotente, […] que no puedes hacer algo así, te puede salir la rabia.”

Participants also held *rabia* responsible for people’s flight behavior that creates some interruption in conflicts, which prevents them from further escalation. While the personal emotion regulation can guide conflictive interactions, these interactions also serve as a form of emotion regulation. Following two interviewees, *rabia* is primarily a feminine emotion that leads opponents to drag more and more people into conflicts. The conversation with third parties who start to react emotionally seems to facilitate the regulation of *rabia*. The degree to which *rabia* can be felt in conflicts apparently depends on factors such as proximity and distance, that is, *confianza* and *respeto*. One interviewee recounted that, unlike in professional relations, she finds it relatively easy to feel *rabia* in personal relations. The dimension of interpersonal distance and proximity seems to be positively correlated with the dimension of low and high intensity of emotionality.

#### Enfado

The interviewees related *enfado* (anger) in so many ways to conflicts that it is impossible to discuss all of its facets here. Some remarks, however, seem important. Some interviewees found it difficult to imagine a discussion without the visceral experience of *enfado*. Apart from the typical relations between *enfado* and different conflict models, *enfado* itself acts as a cultural model of conflict. Almost all interviewees used “enfado” for both an emotion and a conflict model. The difference between both is not always evident because the antecedences of *enfado* as an emotion and as a conflict model are virtually identical.

#### Emotion Regulation

Both emotions and conflicts are tied to interaction dynamics and thus interrelated with each other. A differentiation between forms of emotion regulation within and outside of conflicts emerged from the data.

Interviewees described a variety of ways to regulate emotions outside of conflicts, such as cleaning, sports, mass communions, creative action, conversations with others, and gossip. Gossip with others sometimes leads to the phenomenon that the people entertaining gossip enter a conflict although the original conflict took place between one of the interlocutors and someone else. Interviewees described conversations at work as well as with friends as places where such seemingly cathartic phenomena occur.

The distinction of hot and cold was very common in the interviews and will consequently be used to distinguish between hot and cold ways of emotion regulation within conflicts. Employing a hot way of emotion regulation means to give in to emotions. This is a form of emotion regulation in which individuals actively commit to their emotions. In everyday life language, this active commitment to emotion is sometimes expressed through “soltar las emociones,” which resembles the action of cutting loose a dog. Such upward regulation may be achieved through emulating the expression of emotion, which, assisted by escalating interactions, turns into the expression of real emotions, as one interviewee described.

In many cases, a Freudian hydraulic model of the psyche guides the everyday life conception of emotions in conflict. One participant stated that it is not always good to suppress *enfado* in public, because one would otherwise explode when home alone with his/her conflict partner. Someone else described how it helps her to “relieve tensions” when she just hangs up on someone in the middle of a conflictive phone call. Interviewees also mentioned slamming doors, aggressively placing a bottle on the table, and so on, as forms of emotion regulation with potential effects on the conflict dynamics. Even violence was conceived of as a means of emotion regulation. Interestingly, one participant stated that she used conflicts to regulate her personal emotions. By entering conflicts, she escalates her emotions interactively but subsequently feels relieved.

Cold forms of emotion regulation suppose a more distanced relationship to the experience of emotions and consist in personal control in a stricter sense of the word. An interviewee mentioned the “beso de Judas” as one example. This concept describes how others are treated nicely on the surface only to abuse their trust in order to ridicule them in the next possible occasion. Another example is the emotionally distanced emulation of emotional expressions. In some cases, enfado is only enacted expressively to conform to social expectations. To maintain herself calm in conflict situations, one interviewee takes the perspective of her antagonist who becomes even more *enfadado* when she stays calm, which gives her some relieve from her own *enfado*. Another interviewee knows that apparent indifference can elicit *rabia* in her interlocutors. Under these premises, it results less complicated to substitute hot through cold ways of emotion regulation. Indifference seems to foster similar interaction consequences such as the expression of *enfado* and *rabia*, which might make it easier to employ cold forms of emotion regulation. Other rather cold ways of emotion regulation include the anticipation of vengeance at a later point in time or refraining from benevolent gestures such as bringing lunch for colleagues.

#### Everyday Life Theories of Emotion

Many expressions suggest a psychoanalytical hydraulic or “steam boiler” model of emotions, according to which emotions have to be set free before they create too much pressure. One interviewee indicated with the word “salir” (come out) that she cannot hold back her tears. More personal activity is implied in the word “sacar” that indicates that emotions have to be brought out. The word “soltar” (to release) implies less activity than “sacar” (to dig out/to bring out) but more than “salir” in the process of experiencing and expressing emotions. All three terms were commonly used in the interviews. Alternative forms are “liberar” (to free), “desahogar” (to vent), and “echar balones fuera” (lit. to throw out balls) to describe the reduction of pressure caused by emotions. The hydraulic model is expressed even more clearly through “válvula de escape” (exhaust valve) and “canalizar” (to channel).

The term “tensiones” is often used to refer to the pressure that can supposedly be reduced by engaging in emotional behavior. Boiling blood is a typical image of the pressure character of emotional experience. While the forms of reducing tensions are conceived of as more or less deliberate, the tension is perceived of as building up without personal involvement. The expressions “me entra mala hostia” (bad mood enters), “me da asco” (it gives me disgust), and “me entra tristeza” (sadness enters) indicate that emotions are forced upon individuals and not the product of their own choice. In cases where no “valve” can be found, they might also be suppressed with a certain effort, which is expressed, for example, through terms like “tragar” (to swallow), “oprimir” (to oppress), and “contener” (to contain). One interviewee described a gesture of containment that indicates that emotions are suppressed.

Interviewees depicted most emotions as rather transitory states that get hot very quickly but also cool down fast. Women were depicted as having a rather cold relationship to their emotions, while men would handle their emotions while still hot, which was sometimes considered as more pure or clean (*puro y limpio*)^3^. Hot people were also considered more spontaneous than cold people. Sometimes, however, very hot manners of regulating emotions appeared unhelpful to participants regarding conflict resolution.

Like in the psychoanalytical version of the hydraulic model, interviewees based their reasoning on a biologistic foundation: “[L]os niños son más primitivos, ¿no? Son más limpios, más puros en verdad los sentimientos. Pero para mi tienen […] los mismos sentimientos casi, <casi!, que los adultos. Pero son más puros […]. O sea, tú en un niño ves mucho más claro la envidia, el miedo ae el respeto, la alegría, la amistad. […] Es algo como innato desde pequeño. Si alguna amiga suya tiene algo, tiene envidia porque ya lo quiere tener.” According to this statement, most emotions are innate and can be observed best in their natural and pure form in children.

From this perspective, there is consequently little room for historical variation. Emotions are modeled as constant in time. Only their expressions seem to vary according to gender-specific, ethical, and aesthetical conventions. Participants described emotions as idiosyncratic phenomena that can only be typified, not constituted by language.

### Emotion Theories in Bali

Twenty-five theme clusters have been synthesized from the Balinese data, 11 of them refer to emotions: *benci*, *hormat*, kecewa, *iri*, *kecemburuan*, *jengkel*, *kasihan*, *kesal*, *malu*, *amarah*, and *takut*. The other clusters concern, among other things, the difference of public and private, *ilmu hitam* (black magic), *karma* (as a religious concept), *hati* (liver), authenticity, *memedi* (the devil), and *banjar* (local community).

#### Hormat

*Hormat* is located in the proximity of respect and honor. Some interviewees named people of old age in general and one’s own parents in particular as addressees of *hormat*. Others also included guests, strangers, and ancestors. Unlike *respeto* in the Basque Country where some degree of *respeto* among interlocutors establishes the working foundations and is as such a prerequisite for nonconflictive everyday life interactions, the right to be treated with *hormat* is considered an achievement in Bali. One interviewee stated that a bad *karma* results from not complying with *hormat*-related obligations. Correspondingly, *hormat* bears religious connotations.

Following the interviewees, *hormat* helps to soothe or even prevent conflicts. *Hormat* fosters a friendly and polite conversation style and helps to prevent violence. People who are treated with *hormat* are considered good conflict mediators, as their advice is generally accepted. Especially older people from the *banjar* (local community) or the village are supposed to be addressees of *hormat*. An ascribed lack of *hormat*, however, may lead to sanctions. *Hormat* was treated as both an emotion and the corresponding decent behavior.

#### Iri

The emotion *iri* (envy) was described as triggered by a mother favoring one sibling over another or by the property of others. Sometimes *iri* is treated as a problem that is tackled by religion. Like envy in the capital sin catalogs of medieval Catholicism, *iri hati* (lit. envious liver) is listed among the six depravities (*enam busuk*) that can be cured via the cutting of teeth (*potong gigi*) as part of a rite of passage in the transition to adulthood that has come out of fashion lately. *Iri* is related to the emotion *amarah* (anger).

#### Amarah

“Amarah” is typically translated into “anger.” The antecedents of these emotions seem to be similar indeed. Interviewees mentioned the transgression of norms such as a lack of manners, laziness, deliberately produced loud engine sounds, lying, and so on. Put differently, the disappointment of expectations elicits *amarah* just like the frustration-aggression hypothesis (Bateson, 1941; Miller et al., 1941; Dollard et al., 1971) suggests. Participants also discussed humiliation and spiritual possession as triggers of *amarah*.

Unlike the ethnographic literature suggests, *amarah* does lead to outbursts of expression in conflict situations. Interviewees stated that *amarah* could lead people to throw tools around, shout, rage, and even resort to violence. Outbursts of expression, however, are not very common. The typical way of expressing *amarah* consists in slight modifications of habitual actions. People do not attend meetings, do not converse as freely as usual, and, most prominently, remain silent. Silence seems to be the most conventional way of expressing *amarah*. A matrimonial quarrel coined by *amarah* typically follows the pattern of mutual silence that might every now and then be interrupted by hesitant talk when guests are visiting. Couples eventually start to talk again when their *amarah* has vanished after some days.

Interviewees found it very difficult to suppress *amarah* or to control the related expressions. It was generally considered a tough task to keep *amarah* in the inside or the self (*di dalam diri*) or in the liver (*di dalam hati*). Interviewees stated that it would be impossible to suppress it entirely, as traces of its expressions would involuntarily appear on people’s faces. Some interviewees considered age as an important factor to conduct downward regulation of *amarah*. For that reason, communication and encounters with the addressees of *amarah* are rather avoided whenever possible. There is, however, an emotional antagonist of *amarah* that seems to neutralize it at its onset: *malu*.

#### Malu

Interviewees related how difficult it appears to them to experience *amarah* in public, as the public sphere would elicit an emotional antagonist of this emotion. *Malu* (shame) seems to neutralize *amarah* at its onset as a sociocultural mechanism of emotion regulation that renders deliberate efforts of emotion regulation unnecessary. Particularly in public, *malu* helps to soothe conflicts counteracting the experience of *amarah*. One interviewee stated how he learned to be silent while feeling *amarah* because his outbursts had led him to feel *malu* in the past. *Malu* also interferes in insults that, when occurring in public, do not provoke *amarah* but *malu*.

#### Emosi

When contrasted with the Basque Country, the relations between emotions and conflicts seemed rather hypocognated in Bali. Participants used the term “emosi” primarily as a generic term with negative connotations to refer to nonbenign aspects of conflict. As such interviewees often used it interchangeably with *amarah* or attributed it to the devil (*memedi*). In some cases, it appeared that the interviewees did not try to stay clear of particular emotions but of all emotions during conflicts. Interviewees had trouble relating individual emotion terms to conflicts in general or particular stages of conflict in particular. Instead, they offered often rather general views on emotions without any reference to concrete situations, which sharply contrasts with the elaborate and detailed talk about emotions in conflicts from the Basque Country.

#### Memedi

The term “memedi” refers to the devil or Satan in Balinese. Compared to secular standards, the belief that *memedi* influences in everyday affairs is widely spread in Bali. One interviewee described how *memedi* takes possession of individuals by entering their self (*diri*), causing mental chaos (*kacau*) and craziness (*gila*).

*Amarah* is considered the result of *memedi*’s influence in some cases that are, apart from a general disorientation, not easily discernable, so that *memedi*’s presence can advance to a constant concern to some who might turn to an expert to get assurance about the state of affairs. *Memedi* is supposed to lead people, among other things, to grab a weapon in order to hurt themselves and others. In case of the slightest traces of *memedi*’s emotional manifestations, one participant stated that people would refrain from introspection to avoid contact with alien elements that could interfere with personal thought.

One interviewee gave a rather profane explanation for the assumption that *memedi* preferably appears in the evening. He sees *memedi* as the result of an empty stomach that has to be filled in order to avoid the manifestations of *memedi* in one’s actions. Interviewees stated that most people would simply react with indifference and stay silent when facing emotional outbursts in order to reduce the influence of *memedi* on emotion and conflict.

There are also more elaborate ways to contain the influence of *memedi*. One interviewee talked about a technique of mutual introspection that has to be conducted by an expert in order to avoid grave consequences. Another referred to the ritual practice of employing bloody fetal water to protect the house of a newborn. Regarding such practices, mistakes have to be painstakingly avoided to keep *memedi* away. Offerings and cleaning ceremonies were mentioned as other ways to contain *memedi* and other spirits (*roh–roh*), as well as their emotional manifestations.

#### Emotion Regulation

Considering their mostly moderate and calm habitual expressiveness, Balinese people might appear as experts of expression control at first sight. Closer examination reveals, however, that the underlying techniques concern emotions in the first place, not expressions. Interviewees considered Balinese conflicts as characterized by silence and retreat rather than by direct confrontation. Even the relatively rare occasions of emotional outbursts are followed by a period of silence and retreat. Communication among opponents was described as difficult in conflict situations and is thus avoided. Representatives of the traditional order (*adat*) as well as the state often serve as mediators in conflicts. Both the high status of these mediators and the fact that mediation is often conducted publicly are likely to produce *malu* in the opponents, which neutralizes *amarah* at its onset. Resulting from a particular sociocultural disposition, *malu* acts as a substitute for deliberate efforts of emotion regulation.

Interviewees mentioned many ways to regulate emotions outside of conflict situations. These range from fishing and playing chess to tossing stones, socializing with friends, and creativity. All of these forms concern the emotions and not their expressions. Interviewees related the capacity of emotion control to age and education. Especially children are considered to have difficulty regulating their emotions. One interviewee mentioned the philosophy of Catur Guru, according to which emotions could be tamed with the help of four types of teachers: Guru Rupaka (the parents), Guru Pengajin (school teachers), Guru Wisesa (governmental representatives), and Guru Swadhyaya (god). *Hormat* toward these institutions is supposed to help regulate emotions and conflicts. A dogmatic orientation on traditional hierarchies and representatives of hegemonic belief systems seems to be treated as an alternative to a mature emotionality.

*Hormat* and *malu* appear to be the emotional companions of this rather cognitive orientation on traditional hierarchies and representatives of belief systems. This renders intelligible why for one interviewee praying serves as a means to regulate his emotions. Another interviewee connects the religious aspect of emotion regulation to the communal life in the village (desa). According to this interviewee, the experience of communion is based on the god Wisnu as the preserver of the world, while ceremonies serve as reinforcing agents to soothe conflicts and facilitate emotion regulation. At sacred places such as temples, violence is virtually impossible. These places are regarded as the gates to ancestors who are addressees of *hormat*. It seems that some places trigger *hormat* and thus neutralize emotions like *amarah* and prevent conflicts.

The ritual practice of cutting teeth in males at traditionally 16 years of age is supposed to protect individuals from the six depravities (*enam busuk*). As already mentioned, one of these depravities is the emotion *iri*. The ceremony is considered a rite of passage that marks the transition from childhood to adulthood. The practice has become less popular recently, as it is expensive and time consuming.

#### Authenticity

Talk about authenticity was partly restrained through misunderstandings. Clarifying conversations showed that the notion of, for example, an inauthentic smile did not make sense to some interviewees, as they perceived of emotional expressions as not subjected to voluntary control. Participants stated that only very few people, particularly people of old age, were able to control their expressions. According to the participants, it is very difficult to smile while experiencing *amarah*. And in cases when this is actually achieved, the interviewees expected to see traces of the true emotion or gestures of containment. Participants saw the only feasible way of regulating emotional expressions in overcoming the corresponding emotions through the already mentioned techniques of emotion regulation or through the cultural regulation mechanisms of *hormat* and *malu*.

The notion of authenticity as inner feelings that are not influenced by others seems not to be applicable in Bali. At least, emotions do not appear to belong to the substantial core of the authentic self, as they are envisioned as volatile in nature. In addition to character and temperament, the authentic was identified with the intentional or ideal self or behavior. Surprisingly, it seems to be subject to the will and to be identified with less short-lived phenomena.

#### Everyday Life Theories of Emotion

One interviewee considered emotions as tied to their expressions with little variance in view of situational factors. Interpersonal differences were attributed to personal traits such as the soul (*jiwa*) or problematic states of *jiwa* (mental problems). Another participant depicted the passivity with which emotions are experienced metaphorically. He described emotions as water: While a small stone causes small waves when thrown into water, a big stone causes bigger waves. Unlike the classical ethnographical literature suggests, one interviewee attests Balinese people a very explosive emotion world. For that reason, people would have to be treated with special care during conflicts to prevent them from, for example, weeping.

Metaphors and expressions used during the interviews were very consistent across participants. The term “emosi” (emotion) clearly has a European root. Like “gefühl” in German and “feeling” in English, there is, however, another term that is often used as a synonym for “emotion”: the word “perasaan” and its derivates. Unlike *gefühl* and feeling, *perasaan* does not evoke the notion of haptic but of gustatory sensations. In some Roman languages such as French, for example, “sentir” can refer to feeling an emotion but also indirectly to taste, which is to some extent comparable with the Indonesian metaphor.

Unlike in present-day Europe, however, the emotions are located not in the heart but in the liver (hati) like in ancient Greece. Accordingly, “broken heart” is translated into “sick liver” (*sakit hati*). The reflective localization of emotions in the liver does not necessarily imply that emotions are also prereflectively experienced in this body part like Lakoff (2016) would suggest. Balinese people translate “hati” regularly into “heart” without experiencing any estrangement from the original meaning. On a more general level, interviewees located emotions with the help of a topographical model. Emotions are placed on the inside (of *diri* or *hati*) and either find their way from there to the outside (*keluar*, *mengeluarkan*, *timbul*) or are hidden (*terpendam*), buried (*memendam*), or kept (*menahan, ditahan*) on the inside.

### Emotion Theories in the German Ruhr Area

A total of 32 clusters emerged from the data from the Ruhr Area. Out of these, 16 clusters were concerned with different emotions such as *ärger*, *sauersein*, *traurigkeit*, *angst*, *scham*, *neid*, *eifersucht*, *hass*, *stolz*, and *mitleid*.

#### Ärger

*Ärger* serves as an emotion (anger) and a conflict model. One interviewee distinguished between *streit* (quarrel) and *ärger* by defining *streit* as necessarily overt, whereas *ärger* could also be a latent form of conflict. Apart from this specification, “ärger” and “konflikt” appear to be interchangeable in some contexts. At least one participant considered the emotion *ärger* a component of all forms of conflict. Others viewed anger as typical for different forms of conflict but not as a necessary component.

Just like the main antecedents of anger, *ärger* seems to occur preferably in situations where norms are transgressed. Also disappointed expectations seem to play a role in eliciting *ärger*. *Ärger* is considered a short-lived emotion but, as one interviewee states, can be lasting in the sense that it reemerges in interactions with people the anger is directed at.

Participants find other people’s *ärger* to be justified in some situations, not in others. As a result, the expression of *ärger* is occasionally conceived of as a claim to be right. *Ärger* can consequently become the topic of concern in conflicts, substituting the original issues. The statements of interviewees led to the conclusion that sometimes the conflictive issues can be resolved as soon as an agreement is reached concerning the justification of *ärger*.

#### Vertrautheit and Respekt

One participant discussed *vertrautheit* (intimacy, familiarity) as the reason why he would not quarrel with certain people. In relationships with high levels of *vertrautheit*, it seems to be virtually impossible for him to enter into conflicts due to the amount of *respekt* he experiences in these relationships. This conception differs from the Basque views on *confianza* and *respeto* as opposite poles with differing consequences for conflicts. Unlike the Basque interviewees, German participants considered *respekt* as something to be gained by achievements rather than as a universal base for relationships.

#### Stolz

Participants depicted *stolz* (pride) primarily as a cause of conflicts. Especially *gekränkter stolz* (wounded pride) was treated as a conflictive emotion. Interviewees mentioned insults, disappointed expectations, a lack of recognition, and belittling acts as triggers for wounded *stolz*. Wounded *stolz* was also considered as an obstacle for reconciliation. *Stolz* was sometimes related to masculinity and not always recognized as justified, which some participants held accountable for conflicts. One interviewee described that he considers people who express phony pride as nonpersons or pathologizes their behavior in order to be able to ignore them. Justified *stolz* results from personal achievements and does not bear as negative connotations as unjustified *stolz*.

#### Emotion Regulation

Participants presented many examples of uninhibited expression of emotions in conflicts. This behavior was mainly justified with the help of a steam boiler model, often implied by the term “valve.” One interviewee stated that he tries to dispose of certain emotions through violence just as recommended by his martial arts coach. He justified that by mentioning that he did not want to regret not having let out his emotions and thus having to feel them in future situations.

Not all participants, however, consider direct expressions of emotions the best choice. Two interviewees stated that the verbalization of emotions would sometimes be more effective in conflict situations, as it signals a certain ability to control one’s emotional states, whereas the opponent still struggles to do so and can hence be labeled too emotional to solve conflicts, which places the blame on this person. As a result, the own emotions might become less intense. One interviewee mentioned slander and mobbing as ways to regulate his emotions in conflicts, which would help him to “vent” (*luft ablassen*). Another interviewee devaluates his opponents cognitively or through conversations with others to cope with his emotions. Some interviewees choose retreat from conflicts as a means of emotion regulation. They prefer to be left alone to think about conflict issues rather than communicatively processing them.

Other participants employ the more classical ways of inhibition or camouflage of expressions or apply emotion work. The underlying techniques were often depicted stereotypically. Participants recommended counting to a certain number, taking deep breaths, or employing respiration techniques. Others mentioned “runterschlucken” (to swallow), “verkochen lassen” (to let boil down), physical exercise, making a fist in the pocket, or just “beherrschen” (to keep under control) as ways of emotion regulation. In most cases, interviewees were convinced that there are certain ways that they use to downward regulate their emotions without being able to describe them reflectively.

#### Everyday Life Theories of Emotion

Many expressions suggest that participants used a hydraulic model to conceptualize emotions: “sich Luft machen” (to give vent to something), “in sich hineinfressen” (to bottle up something) “anstauen” (to bottle up one’s feelings), “geladen” (charged), “platzen” (to burst), “das Fass zum Überlaufen bringen” (lit., to bring the barrel to overflow), “angestaute Wut rauslassen” (let out pent-up rage), “Ventil” (valve), and “angestaute Energie” (pent-up energy). A topographical model of inside and outside is often connected to the hydraulic model. This topographical model can, however, also be employed without relation to this model such as in the expression “Wut nach außen tragen” (to carry rage to the outside).

Besides the hydraulic model, descriptions of situations help to depict emotions. Instead of naming particular emotions, interviewees stated they felt “ungerecht behandelt” (treated unjustly), “angegriffen” (attacked), “verarscht” (pranked), or “vernachlässigt” (neglected). These expressions underline that people make use of situations to reflect upon their emotions. Some interviewees found it difficult to find an emotion term and referred either to the situation or people’s intentions to describe emotions.

One interviewee felt that she “could not get through” to her interlocutor, which she equated with the feeling of *traurigkeit* (sadness). For some interviewees, *wut* (rage) is characterized through rising body temperature and an elevated heart rate. One interviewee narrated that she constantly thinks about the trigger of *wut* when she experiences this emotion; another interviewee described that *wut* made her go weak at the knees. One participant recounted that, in one situation, *wut* did not let her sleep until she shouted at her partner, as a result of which she finally fell asleep.

Despite the large variety of linguistic tools to depict emotions, to some participants, emotions remain idiosyncratic phenomena that cannot be reduced to only one dimension: “[Eine Emotion] ist ja immer aus Tausenden von Teilen zusammengesetzt. Eindimensionale Emotionen gibt’s nicht. […] [E]s ist nie der gleiche Gefühlsmischmasch.” The speaker is convinced that each emotion is a unique composite of heterogeneous building blocks, which implies that individual emotions cannot be adequately described by a single emotion term. Other statements suggest that emotions are independent of language.

Some statements are symptomatic for a biologically founded understanding of emotions. One participant attributes the emotional fluctuations his partner experiences to hormonal changes. Apart from biological interpretations, traces of psychiatric, psychoanalytical, and psychological explanations appear in the data. These were inspired by psychotherapy, education, popular culture, and so on. Some emotions, especially seemingly deviant ones, were also attributed to cultural differences such as the allegedly Turkish emphasis on *stolz* that one participant did not consider genuine.

### Cross-Cultural Comparison

The emotions *neid*, *iri*, and *envidia* (envy) seem to evoke negative connotations in all three fields. *Iri* was explicitly related to the concept of six depravities (*enam busuk*). The situations in which these emotions are experienced appear to be similar. Only in Bali, *iri* was more connected to material goods than to interpersonal relations or personal traits. Moreover, the emotion *kecemburuan* (jealousy) was attributed to situations that in the Basque Country would have been probably classified as *envidia*.

*Ärger*, *amarah*, and *enfado* are perceived of as triggering or being triggered by conflicts. They also seem to appear in similar situations. While *ärger* and *enfado* act as both emotions and conflict models, *amarah* was only discussed as an emotion. Participants related *amarah* more directly to violence than *ärger* and *enfado*. Emotions such as *ira*, *rabia*, and *odio* and, respectively, *wut* and *hass* were more evidently related to violence in the other fields. Consequently, *amarah* bears more negative connotations and is less common than *ärger* and *enfado*. *Amarah* seems to be rather hypocognated in Bali and, additionally, serves as a general term to denote various kinds of similar emotions that occur in conflicts. Interviewees considered *ärger* and *enfado* sometimes as a tool for self-protection or to gain respect, which was not the case regarding *amarah*. Although *enfado* and *ärger* are not typically expressed through silence like *amarah*, they are also ascribed to individuals in situations where they do not behave in their habitual ways, with silence being a possible indicator of deviation.

The Balinese data do not contain a conflict-related equivalent of *orgullo* or *stolz*. Both emotions can fuel conflicts, which is particularly obvious in the case of *orgullo*. Interviewees depicted *stolz*, especially wounded *stolz*, as a cause of conflict in the first place, whereas *orgullo* was rather discussed regarding the extension and escalation of conflicts. Both *orgullo* and *stolz* were also considered an obstacle for reconciliation, because both emotions motivate the desire to win conflicts. Unlike what Scheff’s conception of pride suggests, *orgullo* and *stolz* can aggravate conflicts and act as an obstacle to reconciliation. Besides the rather negative aspects of *orgullo* and *stolz*, interviewees also mentioned positive aspects such as ties to personal values. This is particularly true for the Basque Country, where the inside view of *orgullo* received more attention than the inside view of *stolz* in the Ruhr Area, where it was primarily discussed as the wounded *stolz* of others from an external viewpoint.

Participants treated *respeto* as the foundation of human interactions, whereas *hormat* and *respekt* were considered achievements. The data suggest that *respeto* is more reflected upon regarding conflicts when compared to *hormat* and *respekt*. As explained above, interpersonal proximity and distance are emotionally embodied through the axis of *confianza* and *respeto*. In the Ruhr Area, interpersonal distance is rather cognitively codified, although *vertrautheit* bears some similarities to *confianza*. One interviewee from the Ruhr Area stated, however, that *vertrautheit* rather leads him to adopt a calmer conflict style than *confianza* would suggest. *Hormat* seems to have similar consequences as *respeto*, although it does not have a clear opposite and seems to entertain closer ties to general status hierarchies.

*Malu* moderates conflicts, as it neutralizes emotions such as *amarah* at their onset. This is rather surprising from Scheff’s perspective, because unacknowledged shame is supposed to be converted into anger and thus to fuel conflicts instead of moderating them, whereas *malu* seems to be inhibiting certain emotions. Contrary to Geertz’ (1987a) view, *malu*, or rather the Balinese version *lek*, seems not to be translatable into stage fright^4^. “Stage fright” and Geertz’ depiction of Balinese cockfights as deep play imply the practice of acting. It might even appear to outsiders that Balinese people employ a kind of method acting to control their expressions. This is, however, not depicted as such in the inside views in Bali. *Malu* seems to authentically modify the emotions, which renders a cognitive orientation on an expressive order unnecessary. Compared to *malu*, *scham* and *vergüenza* (shame, embarrassment) did not receive considerable attention in the Ruhr Area and the Basque Country. A reason for that might be that emotion regulation in Bali is often conducted via a quasi-automatic social–emotional institution that uses *malu* as its medium, whereas participants from the other two cultural contexts treated emotion regulation more as a deliberate individual or interpersonal endeavor.

In the Basque Country and the Ruhr Area, interviewees often referred to psychoanalysis, psychology, biology, and medicine to justify and legitimize their opinions regarding emotions. This might be based on self-help literature, psychotherapy, education, and/or popular culture. Especially one Balinese interviewee also referred to these rather modern sources of legitimization. Generally, however, more traditional categories such as *dharma* (religious norms), *karma-pala* (karma), *ilmu hitam* (black magic), and so on, were prevalent in the data. Participants also mentioned the epics Mahâbhârata and Ramayana as sources of ethical maxims of feeling and behavior. The data from the Basque Country and the Ruhr Area also bear traces of a religious past such as the biblical “eye for an eye,” but they occupy far less space and seem to be comparatively irrelevant for the everyday life depiction of emotions in conflict.

Despite these differences in interpretation and legitimization, interviewees from all three fields considered emotions as passive phenomena that are relatively transitory. Even in Bali where outsiders often attribute the moderate expressions of emotions to the intentional orientation on a strict expressive order, emotions are portrayed with a hydraulic model. Surprisingly, however, the inhibition of emotional expressions was not at all pathologized regarding health and quality of human relationships as in the Basque Country and the Ruhr Area. This might be due to differences in the conception of authenticity and the fact that emotional processes seem to neutralize other emotional processes as a form of cultural emotion regulation to a greater extent than in the Basque Country and in the Ruhr Area. The variation encountered here is not so much one of the ability to control emotions; it is rather a variation in degree to which the regulation of emotions and expressions is conceived of as being subject to the individual will. Counterintuitively, this degree was lowest in Bali.

Participants from the Basque Country often chose a rhetorical perspective. They depicted their own emotions and expressions as strategic tools to influence the behavior and emotions of others. In the Ruhr Area, participants related emotions to conflicts from a rather distanced perspective, discussing emotions regarding their functions for social entities and individuals. Basque participants were able to relate individual emotion terms in a very reflected way to conflicts, whereas participants from the Ruhr Area employed more descriptions of situations to refer to emotions. The conversational handling of emotions and conflict was comparatively restricted in Bali. Unlike the Basque participants, Balinese interviewees do not seem to cultivate a highly elaborate tradition of interpersonal conflicts. When compared to each other, Bali seems to be more Apollonian, and the Basque Country more Dionysian, in the sense of Nietzsche (1907). The Basque level of reflectivity regarding emotions in conflicts and the corresponding articulate contouring of individual emotions contrast sharply with the obscurity in which emotions and conflicts are left as negatively connoting and rather blurrily recognizable phenomena in Bali.

Although this was most obvious in the Ruhr Area, participants from all three fields relied primarily on their interpretations of situations when identifying emotions. The subtle nuances and omissions of behavior as well as conventionalized signs as silence in Bali were the main points of reference for the identification of emotions. Universal expressions might play a role, although identical expressions do not necessarily carry the same meaning and lead to the same consequences independently of the context. As Plessner (1983) puts it, the chemical analysis of parchment does not contribute to deciphering the meaning of the text that is written on it.

## Discussion

The study bears a number of limitations. The gender ratio in Bali is unbalanced. A woman would have probably gained access more easily to other women in Bali where only one woman was interviewed. The interview with another woman was aborted because of communication problems.

The languages used during the interviews are the national languages Indonesian, Spanish, and German, not the local languages Balinese and Basque. To minimize the effect of language choice, feedback has been obtained from native speakers regarding the translation of emotion terms. Particularly in Bali, this was a concern. The lists of emotion terms were initially very complex in Bali and in Germany and had to be reduced in order to facilitate navigation during the interviews. The interviews required more effort for some interviewees than for others, so that rather complex tasks, questions, and inconvenient topics were left out in some cases.

The method of interpreting narratives from other fields has to cope with the problem that their linguistic form already provides narratives with a certain degree of interpretation. It has been tried to reduce this bias through behavioral descriptions. Instead of talking about greetings, for example, the Spanish practice of greeting through two kisses on the cheeks has been described in detail. This, however, led to another problem in Bali where the two kisses became the scandalous center of attention, which obscured the primary topic of the story, envy.

The study did not make use of video or audio recordings of natural conversations where emotions were processed in conflict situations. Its results, however, can pave the way for an interpretation of such material. In this regard, as well as in four other ways, the study serves exploratory purposes. First, the narratives obtained through the interviews can serve as the base for future research of this kind. Second, more criteria, such as milieu, education, and subcultural belonging, can be taken into account in future research. Third, future studies can get by with a leaner research design, building on the results of this study. Fourth, the study enables researchers to use categories and develop field-specific research questions that individuals find relevant.

Although the interview guide was further developed during the study in view of unexpected evidence from the data, readers might reasonably raise questions of theoretical saturation. Because of a lack of resources, a pragmatic end of data collection and interpretation had to be drawn at some point. More theme clusters could have emerged from the data and could also have been more deeply analyzed with the help of additional interviews. There was definitely a trade-off between depth of analysis of the data obtained in each of the cultural contexts and the comparison of the results. I am confident, however, that the results are faithful to insiders’ viewpoints because the analysis focused on the most prominent and well-outlined clusters and also because I discussed the findings with individuals from the three regions in different occasions. More iterations of data collection and analysis could, without a doubt, produce more insights but would have required a whole team of researchers and more time resources.

The main insights presented here concern culture-specific socioemotional means of emotion regulation as shown with regard to the emotions *malu* and *hormat*, as well as *confianza* and *respeto*. As a result of the sociocultural configuration in Bali, *malu* seems to neutralize emotions such as *amarah* at their onset, acting as a substitute for deliberate forms of emotion and expression regulation. These findings contrast with Geertz’ interpretation of *lek* (i.e., *malu*) as stage fright, which appears to be influenced by his own everyday life theories. Unlike what Geertz suggests, Balinese participants did not interpret their behavior as the result of acting in accordance with a strict expressive order but as guided by unregulated emotions. When compared to the findings concerning *malu* and *orgullo*, Scheff’s concepts of shame and pride as indicators of an insecure or secure bond also seem to be ethnocentrically biased. Instead of being converted into anger, *malu* seems to neutralize similar emotions without any pathological connotation as evoked by Scheff’s treatment of unacknowledged shame. When compared to *orgullo*, in turn, Sheff’s concept of pride does not account for the prolongation and escalation of conflicts that participants in some cases attributed to *orgullo*. It seems to me a promising undertaking to examine socioemotional means of emotion regulation in more detail in different cultural contexts.

The upward regulation of emotions in conflict was particularly present in the data from the Basque Country and should be further examined. Some emotions turned out to serve as frames for cultural models of conflict in the Basque Country and the Ruhr Area. The relations of emotions experienced within these models and the models themselves also deserve further attention in future research. The same is true for the relations among cognitive and emotional representations of proximity and distance in interpersonal contact. Interestingly, emotions have been considered passions in all three fields despite different sources of building blocks for everyday life theories and their legitimization. Surprisingly, the hydraulic model of emotion was used even in Bali. The similarities regarding the models combined with the fact that emotion regulation takes different paths especially in Bali raise questions regarding the mutual influence of linguistic models and social practices. Following Heidegger (1967), everyday life theories do not directly influence prereflective experience. Whether emotions as objects are metaphorically situated in the liver or the heart has no direct influence on prereflective experience or embodiment of emotions like what Lakoff (2016) suggests. Balinese people seem to be able to translate “hati” into “heart” without experiencing any loss of meaning or sense of inadequacy, which may inspire us to rethink our concepts of embodiment.

The design of the study may invite similar cross-cultural research on emotions in conflicts in other cultural contexts. Regarding the regions under consideration in this study, a higher degree of methodological pluralism could be fruitful. The results presented here could be the foundation of the interpretation of audiovisual data from conflicts in natural settings and/or of quantitative studies for comparisons on a bigger scale and precise analyses of the interrelatedness of the emerged categories and dimensions. The future of cross-cultural research on emotions in conflicts would greatly benefit from a methodologically sound triangulation of qualitative and quantitative methods. Regarding cross-cultural research on emotions in general, the study may inspire examinations of the emotions that were related to conflicts here in relation to other common action contexts (such as care settings, education, and counseling) in order to eventually generate a full picture of individual emotion phenomena across different situations.

## Data Availability Statement

The datasets generated for this study are available on request to the corresponding author.

## Ethics Statement

Ethical review and approval was not required for the study on human participants in accordance with the local legislation and institutional requirements. The patients/participants provided their written informed consent to participate in this study. Written informed consent was obtained from the individual(s) for the publication of any potentially identifiable images or data included in this article.

## Author Contributions

RK drafted the manuscript, designed the study and participated in each of its phases, and participated in the review and revision of the manuscript, and has approved the final manuscript to be published.

## Conflict of Interest

The author declares that the research was conducted in the absence of any commercial or financial relationships that could be construed as a potential conflict of interest.

## Footnotes

1 The “Affektives Diktionär Ulm” has been kindly provided by Dan Pokorny.

2 The numbers regarding higher education also include participants who are currently students or have never completed their degrees. For Bali, the numbers are estimates derived from the current professions of the participants and have to be considered the minimum share of participants with higher education.

3 Stereotypes in general and gender stereotypes in particular are devices to talk *about* people and not to interact *with* them, which hints at the fact that stereotypes are not useful to coordinate social action. As a result, social practices are governed by stereotypes only in very few cases, even though participants might agree that they share the same stereotypes. In this particular case, the stereotypes were shared by both male and female participants, the latter of which seemed to stigmatize their own gender. With his distinction of readiness-to-hand and presence-at-hand, Heidegger (1967) provides a theoretical base to differentiate on two levels of analysis between prereflective and emergent social practices on the one hand and their reflexive objectifications on the other.

4 Many Balinese interlocutors confirmed that to them there is no difference between *malu* and *lek*.
